# A Low-Power Hardware Architecture for Real-Time CNN Computing

**DOI:** 10.3390/s23042045

**Published:** 2023-02-11

**Authors:** Xinyu Liu, Chenhong Cao, Shengyu Duan

**Affiliations:** School of Computer Engineering and Science, Shanghai University, Shanghai 200444, China

**Keywords:** CNN, hardware acceleration, edge computing, RTC

## Abstract

Convolutional neural network (CNN) is widely deployed on edge devices, performing tasks such as objective detection, image recognition and acoustic recognition. However, the limited resources and strict power constraints of edge devices pose a great challenge to applying the computationally intensive CNN models. In addition, for the edge applications with real-time requirements, such as real-time computing (RTC) systems, the computations need to be completed considering the required timing constraint, so it is more difficult to trade off between computational latency and power consumption. In this paper, we propose a low-power CNN accelerator for edge inference of RTC systems, where the computations are operated in a column-wise manner, to realize an immediate computation for the currently available input data. We observe that most computations of some CNN kernels in deep layers can be completed in multiple cycles, while not affecting the overall computational latency. Thus, we present a multi-cycle scheme to conduct the column-wise convolutional operations to reduce the hardware resource and power consumption. We present hardware architecture for the multi-cycle scheme as a domain-specific CNN architecture, which is then implemented in a 65 nm technology. We prove our proposed approach realizes up to 8.45%, 49.41% and 50.64% power reductions for LeNet, AlexNet and VGG16, respectively. The experimental results show that our approach tends to cause a larger power reduction for the CNN models with greater depth, larger kernels and more channels.

## 1. Introduction

In recent years, many highly complex algorithms such as deep learning have been widely applied, with the increasingly intensive computations in various computational nodes. CNN is one of the most commonly used deep learning algorithms, specifically applied for objection detection [1,2], image recognition [3,4] and acoustic recognition [5,6,7]. Such applications are often performed at an edge node. However, existing CNN models have a large number of parameters and thus a large number of operations are required. For instance, VGG16 has 138M parameters [3]. For such computationally intensive CNNs, it is important to design domain-specific hardware architecture to increase their computational efficiency, in addition to the software-level optimization techniques such as pruning, distillation and quantization. This is even more significant when implementing CNNs on edge devices or edge sensor nodes, which have limited hardware resources and a tight timing/power/area constraint [8]. In addition, for edge applications with real-time requirements, the data may be streamed in real-time to recursively construct the entire input image, which not only imposes tighter timing constraints due to the non-concurrent availability of the input image, but also increases the conventional accelerator architecture’s power consumption to deal with the redundant data streamed at a different time for the later construction of the input image.

In this paper, we propose a low-power CNN accelerator for edge inference of RTC systems. Specifically, we propose to perform the computations of a CNN in a column-based manner, to realize an immediate computation for the currently available input data. We adjust the process of convolutional operations for column-based data within each convolution kernel, according to the data streaming process in RTC systems and the principle of convolutional operations, where some operations are executed in multiple operating cycles to reduce the utilized hardware resources and its power consumption. We design the hardware architecture in a pipeline for all CNN layers and show that the computational resources of a convolutional layer can be significantly reduced without causing an increase for the overall latency, by using our proposed scheme. The proposed accelerator is implemented by a 65 nm CMOS technology and realizes up to 50.64% power reduction.

The contributions of this work are as follows:

1. We apply a pipeline structure for real-time CNN inference, where the input data is streamed and processed in a column-wise manner to realize the minimal computational time once the complete input image becomes available.
2. We propose a multi-cycle scheme, where all columns related to a convolutional kernel, except for the last one, are processed in multiple cycles to reduce hardware resources and power consumption.
3. Based on the proposed multi-cycle scheme, we design the hardware structures for the typical CNN layers and construct them in a pipeline according to the given CNN architecture, to perform column-wise computations.

The rest of this paper is organized as follows. Section 2 introduces the principle of CNN and state-of-the-art CNN accelerators. Section 3 explains the concepts of CNN operations in RTC, the proposed computational scheme and the architecture. Section 4 presents the experimental results, and Section 5 concludes this work.

## 2. Background

### 2.1. Convolutional Neural Network

CNN is a feed forward neural network, which extracts features from the data in grid patterns, specifically by convolutions. A general CNN contains convolutional and pooling layers to form standard hidden layers, and fully connected (FC) layers as the last few layers to provide the final results for the tasks such as image classification.

**Figure 1:**
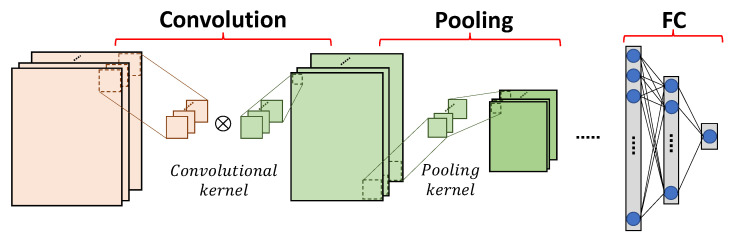
Typical operations of CNN.

We present typical operations of convolutional and pooling layers, as shown in Figure 1. A convolutional layer processes a feature map by sliding a convolutional kernel, also known as a filter, over it. A convolutional kernel performs element-wise multiplications, and produces the result as the sum of all products. The movement of the convolutional kernel over the feature map is defined by the given stride. Therefore, the size of the output feature map of a convolutional layer is determined by the size of the input feature map, the size of the convolutional kernel and the stride. For some CNN models, a convolutional layer consists of multiple channels, where the output feature map is generated by adding up the feature maps of all channels.

A pooling layer is applied for downsampling a feature map, which not only reduces the size of a feature map, and thus reduces the computational complexity, but also increases the resistance against overfitting [9]. Generally, a pooling layer receives the output feature map of a convolutional layer as its input, and produces the result (e.g., maximal or average value) in each pooling kernel. The size of the output feature map of a pooling layer is also determined by the size of the input feature map, the size of the pooling kernel and the stride.

FC layers are usually used as classifiers in CNNs. An FC layer takes the features extracted from the previous convolutional/pooling layers as input and gives the prediction result of the network, where multiple FC layers might be included in a cascade. FC layers limit the network size and produce non-linear combinations of the extracted features.

### 2.2. State-of-the-Art CNN Accelerators

CNNs are both computationally intensive and memory intensive, so CNN accelerators need to be designed and implemented considering the specific applications, based on the trade-off between computational and memory resources. For CNN training and inference, Google proposed the Tensor Processing Unit [10,11,12], which internally employs a systolic array to generate partial results within the array instead of storing them in memory, as a way to reduce the amount of data transmitted between the computational components and the memory. Multiple convolutional layer processors are presented by Shen et al. [13] to perform parallel computations, increasing the computational efficiency and resulting in a higher throughput. These works exploit a high level of computational parallelism, leading to massive computational resources. On the other hand, for some applications with limited computational resources, a CNN can be implemented by utilizing a larger memory to store the intermediate results, with the help of some data reuse schemes to maintain as little memory access as possible. Alwani et al. [14] utilize layer fusion techniques to connect and fuse multiple consecutive convolutional layers of VGG, through multiple buffers, minimizing the number of feature data transferred from the off-chip memory. Wang et al. [15] and Alwani et al. [14] propose to store the intermediate results in the registers on the basis of massive parallelism, reducing data transfer and the requirements of storage space, through data and memory reuse schemes.

For edge devices in RTC systems, the deployment of a large amount of computational or memory resources results in great power consumption, which may not be acceptable depending on the design specifications. In addition, RTC systems usually have strict latency constraints, while their received data streamed over time makes it more difficult to be optimized using the conventional techniques.

Some existing works have provided the strategies to construct the CNN accelerators used in RTC systems. Kim et al. [16] designed lightweight CNNs by quantizing the data from floating-point numbers to fixed-point numbers, and specifically considering the hardware structures, to reduce the overall cost. The same strategy is used in [17], which additionally improves the computational efficiency by the parallism of both convolutional kernels and output channels. Lin et al. [18] propose a Ring Stream Data flow to reduce the amount of data transfer between the chip and the off-chip DRAM, which also uses parallelism to optimize the computational process. A domain-specific CNN architecture is proposed for EEG-based emotion detection, in [19], using a pipeline and parallel hardware architecture, to maximally reuse the computational and memory resources. In [20], a scalable pipeline design is proposed, which also uses layer fusion to reduce the data transfer. A similar architecture can be found in [21]. It can be seen that most of the CNN accelerator designs for RTC take advantage of the natural parallelism of the convolutional computing and run in a pipeline architecture. However, the above work only takes the strict timing and resource constraints into account, but does not consider the recursively streamed input feature map in RTC systems. Even the ring data stream in [18] is only optimized considering a complete input feature map.

In an RTC system, a feature map may be split into different frames that are streamed in at different times. Therefore, the CNN accelerator working in an RTC system adapts the hardware structure and the computational process to this particular data stream. Only a few works present CNN accelerations for this special data stream in RTC: Sanchez et al. [22] propose AWARE-CNN, which converts CNNs to a row-wise computing architecture based on RTC data stream, enabling it to perform computations for a part of feature map when other parts of the input feature map are streamed at the same time. Zhang et al. [23] implement a column-based pipeline architecture to reduce the latency of producing the overall result. However, their works still conduct row-/column-based convolutional operations on the entire convolutional kernel, in a coarse granularity. As the depth of a CNN model increases, the time required to obtain enough rows or columns for the related computations increases accordingly, thus causing great idleness under these approaches. The details will be discussed in Section 3.

## 3. Proposed Architecture for Real-Time CNN

### 3.1. CNN Inference in RTC Systems

**Figure 2:**
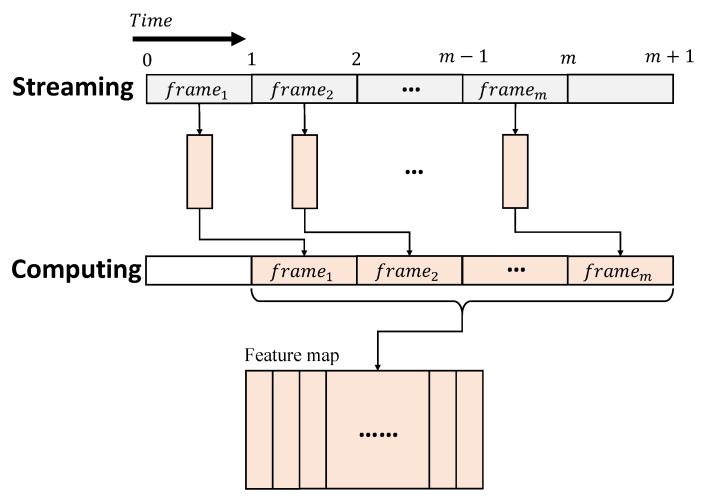
CNN inference procedure in RTC.

In an RTC system, data is streamed as a certain number of separated frames over time, to produce large batches to be processed, as shown in Figure 2. The currently available data is then processed in a strict time limit, often determined by the interval of streaming, to ensure the computation can be immediately initiated, once a stream is received. For a CNN inference in an RTC system, the raw data are treated as an input image, recursively constructed by multiple frames over time. Therefore, the operations, including convolutions and pooling, related to different frames of an input image, are completed at different times. These operations differ from the ones of a conventional CNN, where the entire image can be processed concurrently. A pipeline architecture is therefore suitable to perform a CNN inference in an RTC system. Each pipeline stage works as a specific layer of a CNN and produces the intermediate results, based on the currently available data of the layer. The intermediate results are then propagated to the next stage at the following cycles, so that the input frames received at different times can be processed in parallel [20,21,23].

**Figure 3:**
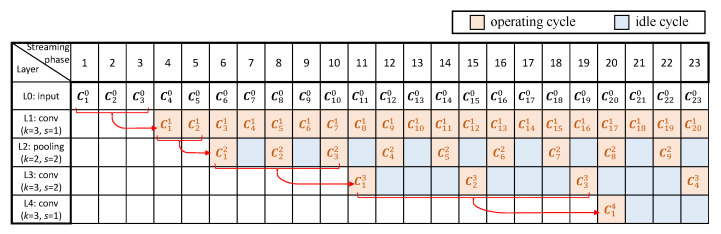
Pipeline timing diagram of an example CNN in RTC systems.

Figure 3 shows a pipeline timing diagram performing the inference of a CNN in an RTC system, which consists of three convolutional layers and one pooling layer, as an example. We give the configuration of each layer, where *k* denotes the size of the kernel and *s* denotes the stride. For simplicity, we assume each column of a complete input image (as a frame) is streamed in at different time period, where we denote $C_{i}^{j}$ as the column data streamed or computed at the *i*-th streaming/computing phase, in the *j*-th layer. Each box around $C_{i}^{j}$ indicates the soonest time when all computations related to $C_{i}^{j}$ can be completed. For instance, the convolutional operations related to $C_{1}^{1}$ can only be performed after the fourth streaming phase in the first convolutional layer because $C_{1}^{0}$, $C_{2}^{0}$ and $C_{3}^{0}$ are involved in such operations (with *k* equal to 3). Note that pipeline stalls might be caused, as some computations may need more lately streamed or computed data in order to be initiated due to the strides of some layers. For example, the pooling operations related to $C_{2}^{2}$ can execute only after the computations of $C_{4}^{1}$ are completed in the previous layer, for the first pooling layer with its stride as 2, which results in a pipeline stall and one idle cycle, before $C_{2}^{2}$ can be processed in this layer.

As shown in Figure 3, idle cycles for some layers are produced because the computations of such layers rely on different number of input frames, causing unbalanced computational time for different layers. This is mostly due to the strides of a CNN. Specifically, for a single-layer CNN, where the kernel size and stride are *k* and *s*, respectively, the computational result of *k* columns’ data is produced for every *s* cycles. We therefore give the duty cycle of this layer, represented by the ratio of the number of operating cycles to the total, as 1/s. For a multi-layer CNN, where all layers are connected in a cascade, assume that the stride of the *j*-th layer is $s_{j}$ and the duty cycle of the previous layer is $L_{j-1}$. Thus, the duty cycle of this layer, $L_{j}$, can be computed as $L_{j-1}/s_{j}$. Recursively for a certain layer, $L_{j}$ can be expressed by the strides of all previous layers and this layer as:

(1) $$L_{j}=\prod_{k=1}^{j}\frac{1}{s_{j}}$$

According to Equation (1), the duty cycle generally decreases for a deeper layer because of the cumulative multiplications related to the strides of all previous layers. Thus, the deeper a layer is located, the longer its idle time would be. This trend can be observed from the first pooling layer and the second convolutional layer (Figure 3).

### 3.2. Multi-Cycle Pipelining for Computational Resource Minimization

In Section 3.1, we provide the assumption that only one column of data needs to be produced and propagated to the next layer, while the rest of the columns can be previously processed. Therefore, some columns can be calculated in advance without waiting for the entire *k* columns to be obtained. Moreover, as the depth of a network increases, the number of columns for the output of a convolutional layer tends to decrease, but the output of a layer can still be considered as a subset of the one when the stride of this certain layer is 1. This is because each column in the input feature map of the layer is calculated at most as many times as the number of weight columns, when the stride is 1. Specifically, for a convolutional layer, assume the stride and the size of the kernel are *s* and *k*, respectively. Given an integer *n*, one column of the output feature map, which is the (n+1)-th column, can be produced by giving the (n·s+1)-th to the (n·s+k)-th columns of the input feature map. The same column data can also be found in the output feature map when the stride is 1. Thus, all the columns in the output feature map, when s≠1, can be found in the output feature map when s=1. In addition, except for the first and last k−1 columns of the input feature map, the convolutional result of each input column with each column of the convolutional kernel are needed to be produced, when the stride of the layer is 1.

**Figure 4:**
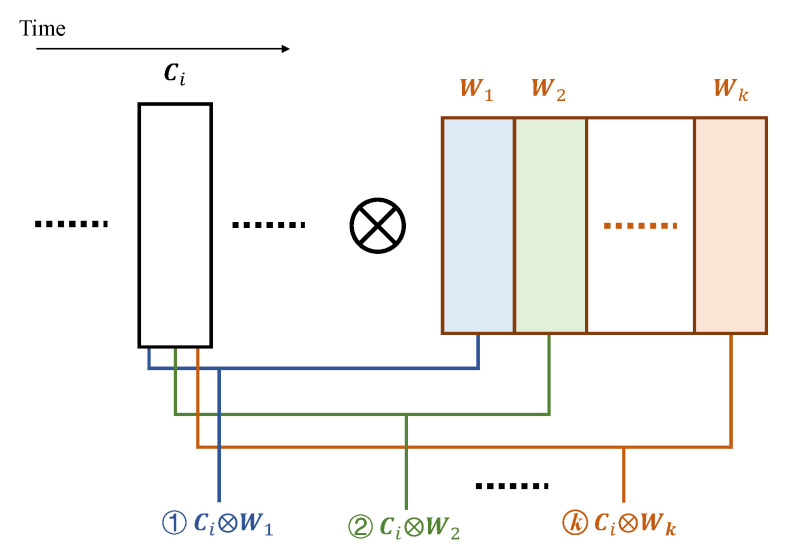
Convolutional operations during one operating cycle.

We propose the multi-cycle pipelining scheme according to the above observation, to reduce the computational resources specifically for convolutional layers. In this scheme, we produce the convolutional result in multiple cycles and the computations related to each column are performed at different cycles. In other words, during each operating cycle for a convolutional layer, we compute the intermediate results for different convolutions related to the same column. In Figure 4, we show all convolutional operations regarding one available column at the moment during an operating cycle.

In order to produce the convolutional result of Equation (2), *k* columns of data are involved, where the intermediate convolutional results ($C_{i-k+1}⊗W_{1}$, $C_{i-k+2}⊗W_{2}$, etc.) are produced over *k* operating cycles. As all operations except $C_{i}⊗W_{k}$ have been completed in the previous operating cycles, the overall convolutional result can be immediately obtained once $C_{i}$ becomes available and $C_{i}⊗W_{k}$ is complete.

(2) $$Conv\left(\left[C_{i-k+1},C_{i-k+2},\cdots ,C_{i}\right]\right)=C_{i-k+1}⊗W_{1}+C_{i-k+2}⊗W_{2}+\cdots +C_{i}⊗W_{k}$$

**Figure 5:**
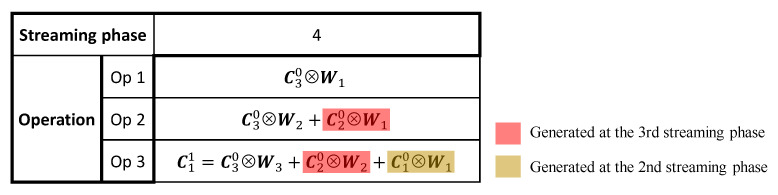
Convolutional operations for the first convolutional layer of Figure 3 at the 4th streaming phase.

In Figure 5, we illustrate all convolutional operations for the first convolutional layer of Figure 3 at the fourth streaming phase, as an example. As can be seen, only the convolutional operations involving $C_{3}^{0}$ are performed in the current operating cycle, and the rest are performed in several previous cycles. It can be noticed that only operation Op 3 is conducted to generate the final convolutional result ($C_{1}^{1}$), while Op 1 and 2 only produce the intermediate results that will be used later. Therefore, only Op 3 needs to be completed in the current operating cycle, in order to ensure the smallest computational latency for RTC systems, while Op 1 and 2 can be completed in multiple cycles, before their produced intermediate results are needed for further computations. In such a case, the hardware resources performing Op 1 and 2 can be reduced based on the number of cycles required to complete these operations, leading to smaller area and lower power consumptions. The above-described multi-cycle scheme is possible because there are idle cycles for some layers during the execution of a CNN in a pipeline architecture, as demonstrated in Figure 3, which can be maximally used to reduce the hardware resources. The number of cycles required for these multi-cycle operations is therefore determined by the duty cycle and the stride of a certain layer, given as $1/(s_{j}\cdot L_{j})$.

**Figure 6:**
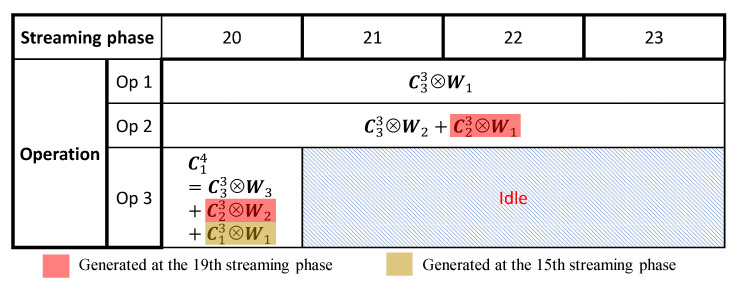
Multi-cycle convolutional operations for the third convolutional layer of Figure 3 during the 20th to 23rd streaming phases.

Figure 6 illustrates the multi-cycle pipeline scheme regarding the operations of the third convolutional layer in Figure 3, during the 20th to 23rd streaming phases.

**Figure 7:**
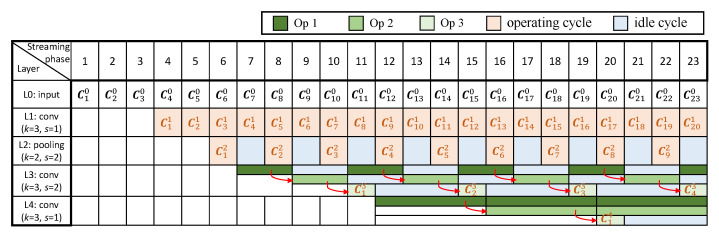
Multi-cycle pipeline timing diagram of the example CNN in RTC systems.

Figure 7 shows the proposed multi-cycle pipeline timing diagram for the inference of the example CNN, as demonstrated in Section 3.1. For the convolutional layers, the original idle cycles shown in Figure 3 are largely eliminated. For the second convolutional layer, the duty cycles of the convolutional operations except the one producing the convolutional result are improved from 0.25 to 0.5. Because the stride of this layer is 2, it is not necessary to perform Op 1 and 2 to all the streamed columns, so there are still some idle cycles. For the third convolutional layer, where the stride is 1, the duty cycles of Op 1 and 2 are increased to 1, which will greatly reduce the hardware resources required to perform these operations, as they can be executed using a smaller circuit running with more operating cycles.

### 3.3. Hardware Architecture

**Figure 8:**
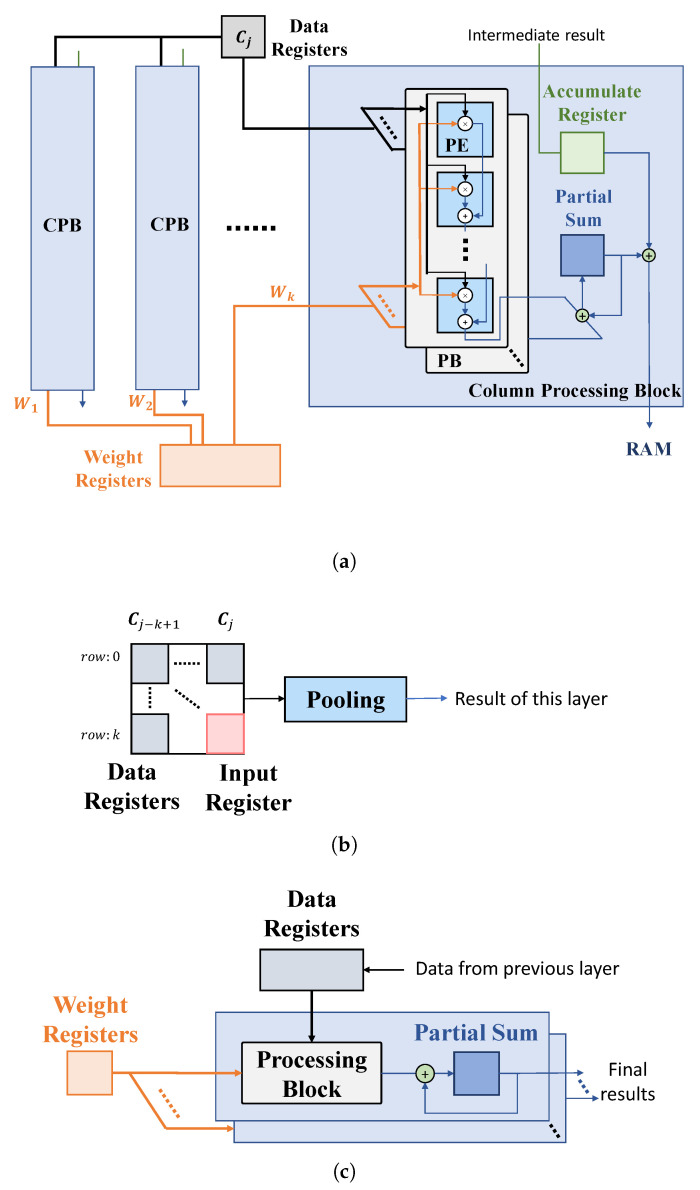
Proposed hardware architecture. (**a**) Convolutional layer. (**b**) Pooling layer. (**c**) FC layer.

Based on the proposed computational scheme in Section 3.2, we provide the structures for the typical layers of a CNN (Figure 8).

(1) Convolutional layer (Figure 8a). Assume that the convolutional kernel includes *k* columns, and thus a convolutional module consists of *k* column processing blocks (CPBs), each performing one column-based convolutional operation, as given in Figure 4. A CPB contains multiple processing blocks (PBs), each processing the computations of an input channel. Note that the number of PBs included in each CPB can be adjusted according to the given design specifications of power, timing, etc., and the final result of a layer can be produced by iteratively processing some input channels in each period. Each PB consists of *k* processing elements (PEs) for a multiply–accumulate (MAC) operation.

During an operating cycle, a CPB firstly fetches the column data produced by the previous layer, $C^{j}$, from the data registers, and the intermediate results of several convolutional operations performed in the previous operating cycles (such as Op 1 and 2 in Figure 7), from an accumulate register. Each PE of a PB performs MAC for an input feature with the corresponding weight and propagates the result to the next PE, until the final result of a column and of an input channel is produced. The results of all PBs are accumulated to produce a partial sum at one clock cycle, while the final convolutional result of a column with all input channel is produced over multiple clock periods, if the number of input channel for a layer is larger than the number of PBs implemented in the CPB. For all output channel, the convolutional results can be obtained by executing the process above, recursively. The final results of the layer can be obtained by adding the single-column convolutional result with the data stored in the accumulated register. Both the final results and the intermediate results of some convolutional operations are then stored in RAM for further computations.

(2) Pooling layer (Figure 8b). A pooling module is typically connected with the output of a convolutional module. This module stores the convolutional result of the previous layer into an input register and fetches the remaining $k^{2}-1$ elements of a k×k pooling kernel, from RAM into the data registers. The pooling block generates the pooling result according to the specific operations, determined by the pooling methods of a certain CNN model.

(3) FC layer (Figure 8c). An FC module is also composed of PBs as the convolutional layer. Each PB represents a neuron, and the number of PBs is determined by the number of neurons. The last FC module gives the final results of a CNN.

**Figure 9:**
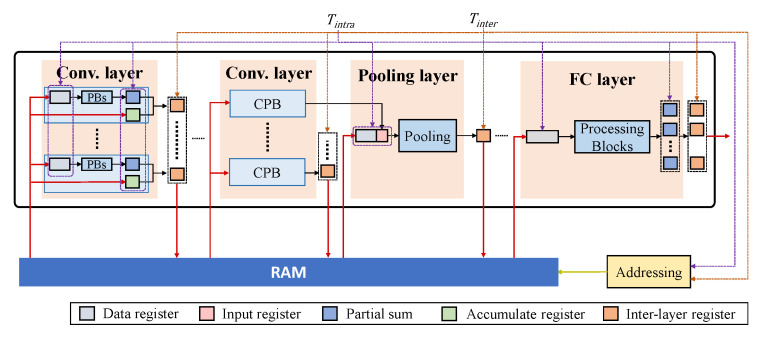
The overall pipeline architecture.

The overall pipeline architecture using the above-mentioned hardware modules is given in Figure 9, where all layers are connected with a RAM. An intra-layer clock with a period of $T_{intra}$ controls all registers within a layer. The convolutional and FC layers operate in a three-stage pipeline as fetch, execution and write back. The result of a layer is produced in multiple clock cycles and stored in the inter-layer registers. The pooling module is typically connected to the output of a convolutional module and, more specifically, to the CPB that produces the final convolutional result of the layer. Although the duty cycle of each convolutional layer is different, each convolutional layer produces the intermediate or final results for at least each streaming phase. Therefore, we use the period of a streaming phase as $T_{inter}$. In such a case, the latency of each layer has to be no longer than $T_{inter}$, to ensure that all currently received data are processed before new input data arrives. Under the above timing constraint, the number of PBs in each CPB can be adjusted to trade off between the hardware resources and the overall computational latency, which determines the number of clock cycles required to produce the result propagated to the next layer. Specifically, assume the ratio of $T_{inter}$ to $T_{intra}$ is *N*, and *N* determines the maximal number of clock cycles that each PB computes to generate the final result. Therefore, if the total number of clock cycles that one PB needs to complete the convolutional computation of a column in a layer is *M*, the number of PBs allocated in the layer should be M/N and rounded up. In addition, the intra-layer and inter-layer clocks are used to address the RAM. Specifically, the intra-layer and inter-layer clocks determine the frequencies, under which the read and write addresses of the RAM are changed.

## 4. Results

### 4.1. Setup

To evaluate our proposed method and architecture, we implement several typical CNN architectures with 8-bit fixed-point quantization by TSMC 65 nm CMOS technology. These designs are synthesized by Synopsys Design Compiler (DC), and we perform static timing and power analysis of DC to measure the clock frequency and the power consumption, under the nominal case of the given technology.

We implement LeNet [24], AlexNet [25] and VGG16 [3] for further evaluation, and provide the configurations and structures in Table 1 and Table 2, respectively. It is worth mentioning the proposed technique is applicable when implementing any CNN models on hardware. In addition, this method is also independent of applications. Thus, some configurations of the models, such as the number of output neurons, may vary due to the different tasks that they are deployed for, but it is always possible to construct different hardware structures with our technique for a given model, considering the trade-off between hardware resources and timing.

### 4.2. Overall Power Reduction

**Figure 10:**
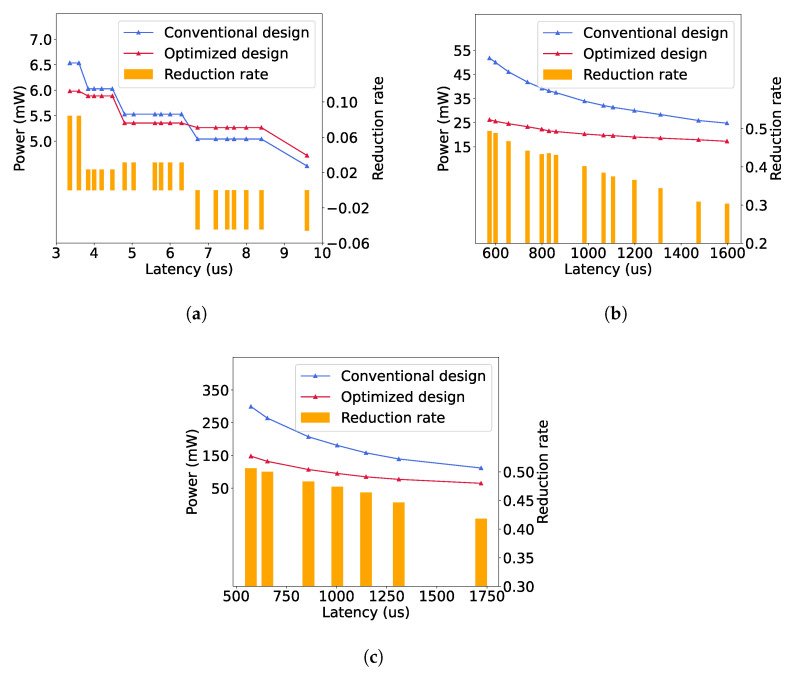
Power consumption and reduction rates. (**a**) LeNet. (**b**) AlexNet. (**c**) VGG16.

We measure the power consumption for each CNN architecture, implemented with a different number of PBs to adjust the computational latency, as described in Section 3.3. Figure 10 shows the power consumption and reduction for each implemented CNN architecture by using our proposed multi-cycle pipelining scheme. Our approach realizes up to 8.45%, 49.41% and 50.64% power reductions for LeNet, AlexNet and VGG16, respectively. Therefore, the power reduction is highly related to the structures of a CNN model. Generally, our approach leads to a higher power reduction for a deeper CNN. This is because the duty cycle of a deeper layer is produced by the cumulative multiplications of the strides of all previous layers, as given in Equation (1), so the computations of a deeper convolutional layer can be completed generally in more operating cycles by using our approach, resulting in fewer hardware resources and less power.

Additionally, Figure 10 indicates that the overall power reduction tends to decrease when the computational latency is increased. This is because the increase in computational latency is caused by fewer PBs allocated in each layer, so the proportion of the circuit where the power can be reduced by our approach (such as Op 1 and 2 in Figure 7) becomes smaller, as the hardware resources of the rest (such as Op 3 in Figure 7) cannot be reduced in order to maintain the same computational latency as that of the original design. However, even in the cases of the maximal latency, our approach reduces the power by 30.36% and 41.81% for AlexNet and VGG16, respectively.

In Figure 10a, it can be noticed that when the latency is greater than 6.2 us for LeNet, the power consumption using our method is greater than that of the conventional design. This is because the number of PBs is the same in both architectures when the duty cycle of a convolutional layer is one, but compared to the original architecture, our approach introduces some extra registers and MAC operations. In addition, since LeNet has only two convolutional layers, the power reduction in the second layer is significantly smaller than the power increase in the first layer, as we will show it in Section 4.3. Therefore, for the networks with few convolutional layers and small amount of computations, our method may fail to reduce the power, especially when its computational latency is high. However, the overall power of a small and slow CNN hardware architecture is generally small, so it might not be necessary to further reduce its power consumption.

We compared the proposed architecture with some existing works, in Table 3, where both works [22,23] present hardware implementations for VGG16, so all the results of our work are measured for the case of VGG16. As has also been described in Section 2.2, both designs [22,23] are proposed to operate in RTC systems, so they have the same inference process as our work. Even though the implementations, technology nodes and quantization methods are different, all the works shown in Table 3 have near results in terms of frequency and latency, as they are all designed with column-based pipeline architectures. The overall power consumption and latency of our work are adjustable by including a different number of PBs into the hardware, so it is possible to trade off between latency and power. We do not compare the overall power consumption, as it is highly related to the specific implementation and the total number of PEs. Instead, we compute the power efficiency in the unit of trillion operations per second per Watt (TOPs/W). It is noticeable that the power efficiency of our design is in the range of 3.19–4.08 TOPs/W, which is higher than the existing works for at least one magnitude. This is because our proposed multi-cycle pipeline scheme can largely reduce the power consumption while keeping the same latency, as shown in Figure 10.

**Figure 11:**
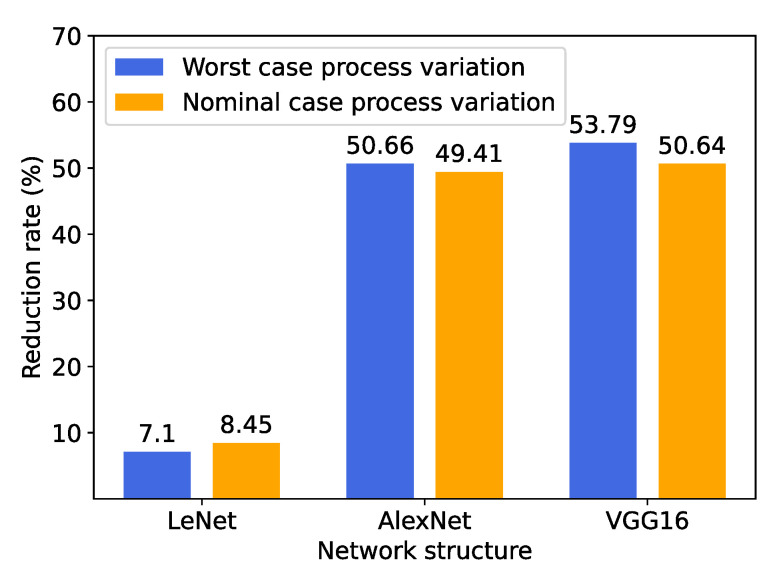
Power reduction rates for the circuits constructed by gates with worst- and nominal-case process variations.

Finally, we re-synthesize the three models by using the logic gates with the worst-case process variations. For each case, we construct the circuit to ensure it has the smallest latency, as in the nominal case shown in Figure 10. We measure the power reduction rates in the worst case and compare them with those of the nominal case, in Figure 11, where it can be noticed that the power is reduced by a similar percentage in both cases for each network structure. According to our evaluation, the overall power consumption in the worst case is around three times the nominal case. This is because the gates with the worst-case process variations are slow so that they are oversized during the synthesis process to satisfy the same clock period as the nominal case, resulting in greater power. The result indicates that our proposed technique can generally lead to consistent power reduction considering different ways to construct a certain model. Therefore, we further conclude that the power reduction is mainly determined by the structure of the CNN model, which is specifically investigated in the following section (Section 4.3).

### 4.3. Impact of Layer Configurations to Power Reduction

**Figure 12:**
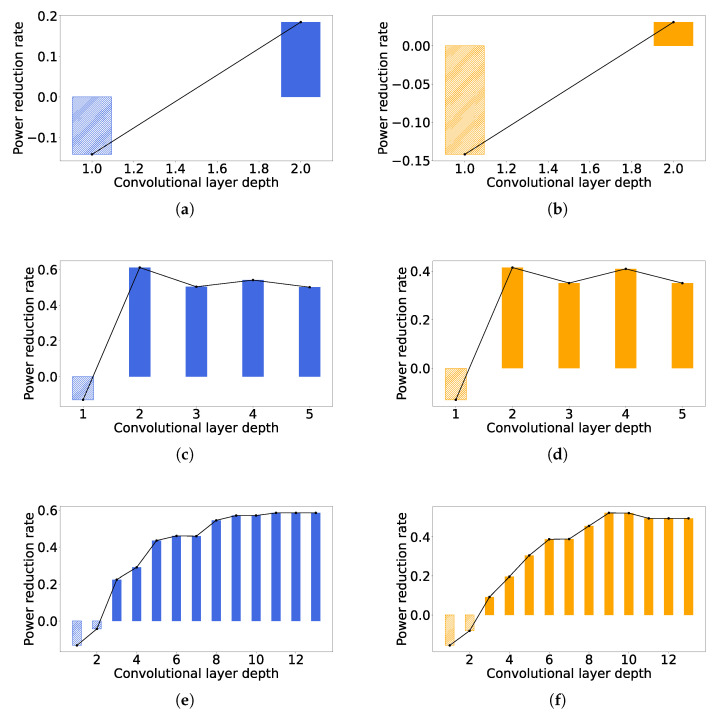
Power reductions of all convolutional layers. (**a**) LeNet, latency = 3.36 us. (**b**) LeNet, latency = 9.6 us. (**c**) AlexNet, latency = 573.4 us. (**d**) AlexNet, latency = 1597.4 us. (**e**) VGG16, latency = 573.4 us. (**f**) VGG16, latency = 1720.3 us.

Figure 12 shows the power reduction for each convolutional layer in different networks implemented with the lowest and the highest latency. By comparing Figure 12a,c,e with Figure 12b,d,f, respectively, it can be seen that the trend of the power reduction with the layer deepening is similar for the same CNN architecture with different latency; however, in the case of high latency, the power reduction of each layer is smaller than that of the low latency case, which is consistent to what we show in Figure 10. It is noticeable from Figure 12 that more power consumptions are produced for the shallow layers, including the first layers of LetNet and AlexNet and the first two layers of VGG16. This is because our method may include more registers in order to store more intermediate results, while the number of computational units maintains the shallow layers, causing greater power. However, for deeper layers, the number of computational units are greatly reduced because some computations can be completed in more cycles to trade off for smaller area and power, and thus the area and power reductions of computational units would compensate for the overhead caused by the extra registers.

We provide some observations for the case of AlexNet from Figure 12c,d, as follows. The power reductions of the second and fourth layers are higher than those of the third layer. The size of the convolutional kernel of the second layer is larger than that of the third layer, and thus the power reduction in the second layer is theoretically larger than that of the third layer because more convolutional operations can be performed in the multi-cycle scheme. On the other hand, the size of the convolutional kernels of both the third and fourth layers was three, and thus the power reductions were expected to be same. However, the fourth layer had more input channels than the third layer, as shown in Table 1, which leads to more PBs allocated to satisfy the timing constraint, so it again causes more hardware resources to be operated in the multi-cycle scheme. The above observation illustrates that our approach tends to achieve a higher power reduction for the convolutional layers with larger convolutional kernels and more input channels.

For VGG16, all the convolutional layers have their kernel in the same size, as demonstrated in Table 1. However, as it goes to a layer with greater depth, the power reduction becomes larger, until it becomes generally constant, which can be defined by (k−1)/k. This is because for a convolutional kernel with *k* columns, the computations for the first k−1 columns can be conducted with our multi-cycle scheme. For a deeper layer, the duty cycle keeps increasing, causing nearly negligible power consumption for the first k−1 columns’ computations. It is also noticeable that the power reduction of the eleventh layer is smaller than that of the tenth layer, as shown in Figure 12f. The reason is that there is a pooling layer between these two convolutional layers, which makes the feature map of the eleventh layer smaller than that of the tenth layer, and makes the eleventh layer less computationally intensive. We can generally conclude that our approach can reduce up to (k−1)/k power consumption, for a convolutional layer with its kernel size of k×k, especially in a deep CNN. Although we only demonstrate the power consumption under the nominal case of a CMOS technology, the theoretical maximum power reduction will not change if the architecture is implemented in the best or worst case, as we have confirmed in Figure 11.

## 5. Conclusions

CNN is one of the widely used deep learning algorithms, often deployed on edge devices with limited resources. The intensive computations and the large storage space required with CNN pose a challenge on its deployment in edge applications. In addition, for the edge applications with real-time requirements (i.e., RTC systems), input data are often split into a certain number of frames, streamed at different times and recursively construct a complete input image. The computational process of CNN for RTC therefore differs from the one used in non-real-time systems. Thus, domain-specific hardware architecture to perform CNN in RTC systems is necessary.

In this paper, we propose a low-power CNN accelerator for edge inference of RTC systems, based on a multi-cycle scheme. This scheme is presented to complete the operations related to all columns, except for the last one in a convolutional kernel in multiple cycles—the number of which can be determined by the depth of a certain layer and the strides of all previous layers and this layer, so that the hardware resources related to these multi-cycle operations can be largely reduced to lower the power consumption. Furthermore, we design hardware modules for the typical CNN layers and present a pipeline architecture to support the above-mentioned operations. The proposed accelerator is implemented by 65 nm CMOS technology. The results show that the power reduction of the proposed architecture increases with the decrease of latency. Our technique realizes up to 8.45%, 49.41% and 50.64% power reductions for LeNet, AlexNet and VGG16, respectively. Moreover, considering a certain CNN model, the power reduction of different layers increases with the depth of the layer. For a deep layer of a CNN with its kernel size as k×k, our approach can theoretically reduce the power to 1/k. Thus, it is suggested to use this approach for the CNN models with great depth, large kernel size and many channels.

## Figures and Tables

**Table 1:** Implemented CNN architectures.

Network	Size of Input Image	No. of Classes	No. of Parameters
LeNet	32×32	10	0.05 M
AlexNet	224×224	21	41.59 M
VGG16	224×224	21	117.57 M

**Table 2:** Structures of the implemented CNN architectures (in: number of input channels; out: number of output channels; k: kernel size; s: stride).

Layer	LeNet	AlexNet	VGG16
conv.	in = 1, out = 6, k = 5, s = 1	in = 3, out = 96, k = 11, s = 4	in = 3, out = 64, k = 3, s = 1
conv.			in = 64, out = 64, k = 3, s = 1
pooling	k = 2, s = 2	k = 3, s = 2	k = 2, s = 2
conv.	in = 6, out = 16, k = 5, s = 1	in = 96, out = 256, k = 5, s = 1	in = 64, out = 128, k = 3, s = 1
conv.			in = 128, out = 128, k = 3, s = 1
pooling	k = 2, s = 2	k = 3, s = 2	k = 2, s = 2
conv.		in = 256, out = 384, k = 3, s = 1	in = 128, out = 256, k = 3, s = 1
conv.		in = 384, out = 384, k = 3, s = 1	in = 256, out = 256, k = 3, s = 1
conv.		in = 384, out = 256, k = 3, s = 1	in = 256, out = 256, k = 3, s = 1
pooling		k = 3, s = 2	k = 2, s = 2
conv.			in = 256, out = 512, k = 3, s = 1
conv.			in = 512, out = 512, k = 3, s = 1
conv.			in = 512, out = 512, k = 3, s = 1
pooling			k = 2, s = 2
conv.			in = 512, out = 512, k = 3, s = 1
conv.			in = 512, out = 512, k = 3, s = 1
conv.			in = 512, out = 512, k = 3, s = 1
pooling			k = 2, s = 2
fc	in = 400, out = 120	in = 9216, out = 4096	in = 25088, out = 4096
fc	in = 120, out = 84	in = 4096, out = 4096	in = 4096, out = 4096
fc	in = 84, out = 10	in = 4096, out = 21	in = 4096, out = 21

**Table 3:** Comparisons between the proposed architecture and the existing works.

Design	AWARE-CNN [22]	ISCAS21 [23]	This Work
Implementation	FPGA	FPGA	ASIC
Technology node (nm)	16	28	65
Quantization (bit)	16	8	8
Frequency (MHz)	30–40	200	62.5
Power (mW)	15,000	NA	65.05–147.95
Power efficiency (TOPs/W)	0.25–0.49	0.045	3.19–4.08
Latency (ms)	30.3–45.2	27.78	7.45–22.36

## Data Availability

All source code and data supporting this paper are openly available at https://github.com/Croyyin/A-Low-power-Hardware-Architecture-for-Real-time-CNN-Computing.git (accessed on 25 October 2022).
